# Molecular surveillance of *Plasmodium falciparum* resistance to artemisinin-based combination therapies in the Democratic Republic of Congo

**DOI:** 10.1371/journal.pone.0179142

**Published:** 2017-06-08

**Authors:** Dieudonné Makaba Mvumbi, Thierry Lengu Bobanga, Jean-Marie Ntumba Kayembe, Georges Lelo Mvumbi, Hippolyte Nani-Tuma Situakibanza, Françoise Benoit-Vical, Pierrette Melin, Patrick De Mol, Marie-Pierre Hayette

**Affiliations:** 1 Biochemistry and Molecular Biology Unit, Department of Basic Sciences, School of Medicine, University of Kinshasa, Kinshasa, Democratic Republic of Congo; 2 Department of Clinical Microbiology, University Hospital of Liege, Liege, Belgium; 3 Department of Parasitology and Tropical Medicine, School of Medicine, University of Kinshasa, Kinshasa, Democratic Republic of Congo; 4 Department of Internal Medicine, School of Medicine, University of Kinshasa, Kinshasa, Democratic Republic of Congo; 5 CNRS, LCC (Laboratoire de Chimie de Coordination), Toulouse et Université de Toulouse, UPS, France; Université Pierre et Marie Curie, FRANCE

## Abstract

Malaria is a major public health problem in the Democratic Republic of Congo. Despite progress achieved over the past decade in the fight against malaria, further efforts have to be done such as in the surveillance and the containment of *Plasmodium falciparum* resistant strains. We investigated resistance to artemisinin-based combination therapies currently in use in Democratic Republic of Congo by surveying molecular polymorphisms in three genes: pfcrt, pfmdr1 and pfk13 to explore possible emergence of amodiaquine, lumefantrine or artemisinin resistance in Democratic Republic of Congo. This study essentially revealed that resistance to chloroquine is still decreasing while polymorphism related to amodiaquine resistance seems to be not present in Democratic Republic of Congo, that three samples, located in the east of the country, harbor Pfmdr1 amplification and that none of the mutations found in South-East Asia correlated with artemisinine resistance have been found in Democratic Republic of Congo. But new mutations have been identified, especially the M476K, occurred in the same position that the M476I previously identified in the F32-ART strain, strongly resistant to artemisinine. Antimalarial first-line treatments currently in use in Democratic Republic of Congo are not associated with emergence of molecular markers of resistance.

## Introduction

In the Democratic Republic of Congo (DRC) malaria is still a major public health problem. Interventions conducted the last ten years led to reduce malaria related morbidity-mortality [1], but further efforts have to be done to reach the aim fixed by the World Health Organization (WHO) to reduce malaria mortality and incidence by 90% in 2030 [2].

Efforts must be strengthened in many topics, such as surveillance and containment of *Plasmodium falciparum* resistant strains. Resistance, that could be defined as the ability of a parasite strain to survive or multiply in the presence of drug concentrations that normally kill parasites of the same species or prevent their multiplication [3], is a phenomenon that has always existed since drugs started to exert pressure phenomenon. All drugs submitted to the malaria parasite became, some years after their introduction, ineffective [4]. In response to it, WHO experts have recommended to use combination therapies including an artemisinin component instead of using monotherapies [5]. Currently, all endemic countries have officially adopted artemisinin-based combination therapies (ACT) as first-line treatment for non-severe malaria.

In DRC, two ACTs have been accepted as first-line treatment by the National Malaria Control Program (NMCP): Artesunate-Amodiaquine in 2005 followed by Artemether-Lumefantrine in 2010. Both combinations are simultaneously in use in the country.

Resistance to previous antimalarials has led to an increase of malaria mortality from 1980 to 2004 [6], an increase of the global cost for disease control [7, 8], and an increase of transmission [9].It is clear that the spread of artemisinin resistance from Asia to Africa would seriously threaten malaria control [10]. This resistance has been clearly established in South-East Asia but currently, no report of artemisinin resistance has been described in Africa [11].

Monitoring parasite resistance to antimalarial drugs is mandatory in malaria control strategies. Molecular markers are a good alternative to *in vivo* treatment trials or *in vitro* drug susceptibility testing to detect drug-resistant strains as they allow analyzing large sample and assessing resistance to many antimalarial drugs simultaneously [12]. Unfortunately, molecular studies require expensive infrastructures and reagents and also qualified persons that are not always available in sub-Saharan Africa. This can explain the small number of studies conducted in DRC [13].

Some molecular markers associated with malaria resistance have been clearly described. The K76T mutation occurring on the *Plasmodium falciparum* chloroquine-resistance transporter (*pfcrt*) gene is the key element of chloroquine resistance [14] and the SVMNT haplotype, defined by specific mutations at amino acid positions 72–76, found in the same gene has been clearly linked to amodiaquine resistance [15]. Moreover, Ariey et al. provided the link between mutations in the propeller region of the *Kelch* 13 gene (K13, PF3D7_1343700) and artemisinin resistance [16]. Mutations on the *Plasmodium falciparum* multidrug resistance 1 (*pfmdr1*) gene and increase of its copy number have been related to resistance to many monotherapies [17–20] but also linked to ACT introduction [21, 22].

In previous studies, we have detected a high prevalence of *pfcrt* mutants in Kinshasa [23] so in the present work we have extended the analysis to five other geographic areas in DRC. We investigated resistance to ACT currently in use in DRC by surveying molecular polymorphisms in three genes: *pfcrt*, *pfmdr1* and *pfk13* to explore possible emergence of amodiaquine, lumefantrine or artemisinin resistance in DRC.

## Materials and methods

### Ethical considerations

The protocol and the informed consent received the ethical approbation from the Ministry of Public Health of the DRC and from the Institutional Committee of the Faculty of Medicine, University of Kinshasa. All the participants involved in the study (or the parents/guardians of children) provided a written consent.

### Study sites and participants

We conducted this study in six areas with different dynamics of transmission: Bolenge, Luzizila and Mweka in the equatorial facies; Punia and Kapolowe in the tropical facies and Butembo in the mountain facies. Malaria transmission is perennial in the equatorial and tropical facies but seasonal in the mountain one. In each site, one hundred individual has been randomly selected in a household survey (except for Punia where only eighty individuals could be selected). The survey was conducted between March and November 2014.

### Blood collection and parasite identification

For each individual, blood samples were collected from finger prick and DNA was extracted by using the QiaAmp^®^ DNA mini kit (Qiagen, Hilden, Germany) as previously described [23].

One real-time PCR consisting in two duplex reactions (identifying *P*. *falciparum* + *P*. *ovale* and *P*. *malariae* + *P*. *vivax*) was run to detect *Plasmodium* species [24] on a Lightcycler 480 instruments (Roche^®^) in the Clinical microbiology Unit of the University Hospital of Liege, Belgium. *P*. *falciparum* positive samples were stored at −20°C for further molecular analysis.

### Assessing *Pfmdr1* copy number

A relative quantification multiplex real-time PCR was run by using a couple of primers plus a FAM-labelled probe for *pfmdr1* detection and another couple of primers with a VIC-labeled probe for β-tubullin detection [25]. Assays were run on a Lightcycler 480 instrument (Roche^®^) in the presence of one single copy control reference strain 3D7. The results were analyzed by the comparative ΔΔCt method. Parasites were considered to have an amplified *pfmdr1* gene if copy number was > 1.5 [26].

### Assessing *Pfcrt* 72–76 haplotypes

A conventional PCR was run to amplify an approximately 152 pb fragment on the *pfcrt* gene containing the region of interest followed by sequencing of amplicons, as described in a previous work [23]. Both 3D7 and K1 reference strains, respectively sensitive and resistant to chloroquine, were used as control during the assays.

### Assessing polymorphisms on the K13 propeller gene

Another conventional PCR was run by using primers recently described by Ariey et al. [16] to amplify a fragment including positions where mutations related to artemisinin resistance were found. All PCR assays were run in the presence of the F32-ART reference strain provided by Centre National de la Recherche Scientifique—CNRS, France. All amplicons were purified on a Sciclone G3 Automated Liquid Handling Workstation (Perkin Elmer, USA) using AgencourtCleanSEQ^®^ kit (Agencourt Bioscience, USA) and then sequenced jointly in the GIGA centre of University of Liege, Belgium and in the Molecular biology platform of the University Hospital of Liege, Belgium using a 3130xl DNA sequencer (Applied Biosystems, USA).

The K13-propeller SNPs were analyzed by comparing with the reference 3D7 strain (PF3D7_1343700) using Sequencher^®^ Software Ver. 5.4.5 (Gene Codes corporation, Michigan, USA) and the online BLASTx tool (National Center for Biotechnology Information, USA).

## Results

Out of the 580 samples collected over the six geographic sites, 280 (48.2%) were PCR-positive to *P*. *falciparum*, among which 6 (2.14%) were mixed infections (combined only with *P*. *malariae*). Distribution of these prevalences by area and by age group has been described in a previous published study [27]. All results obtained for these molecular markers are presented in Table 1.

**Table 1:** Analysis of molecular markers related to Pf resistance in DRC.

		Bolenge		Mweka		Kapolowe		Luzizila		Butembo		Punia	
Mol. marker	Status	N	N (%)	N	N (%)	N	N (%)	N	N (%)	N	N (%)	N	N (%)
*pfcrt*		51		31		63		62		22		51	
	W		15 (29.4)		10 (32.3)		22 (34.9)		24 (38.7)		6 (27.3)		24 (47.1)
	M		36 (70.6)		21 (67.7)		41 (65.1)		38 (61.3)		16 (72.7)		27 (52.9)
pfmdr1*		51		31		63		62		22		51	
	W		51 (100.0)		31 (100.0)		63 (100.0)		62 (100.0)		19 (86.4)		51 (100.0)
	M		0 (0.0)		0 (0.0)		0 (0.0)		0 (0.0)		3 (13.6)		0 (0.0)
*K13*		51		31		63		62		22		51	
	W		50 (98.1)		29 (93.5)		61 (93.7)		59 (95.2)		21 (95.4)		51 (100.0)
	M		1 (1.9)		2 (6.5)		2 (3.2)		3 (4.8)		1 (4.5)		0 (0.0)

### *Pfmdr1* copy number

*Pfmdr1* analysis was successful for all *P*. *falciparum* positive samples. Only three samples (1.07%), all found in Butembo (Nord-Kivu province) have a copy number amplification beyond 1.5.

### *Pfcrt* haplotype

One hundred and seventy-nine samples (63.9%) harbored the 76T mutation among which two had the relatively rare CVMNT haplotype (in Mweka and Kapolowe) and the rest got the CVIET one.

### *Pfk13* propeller polymorphism

**Fig 1:**
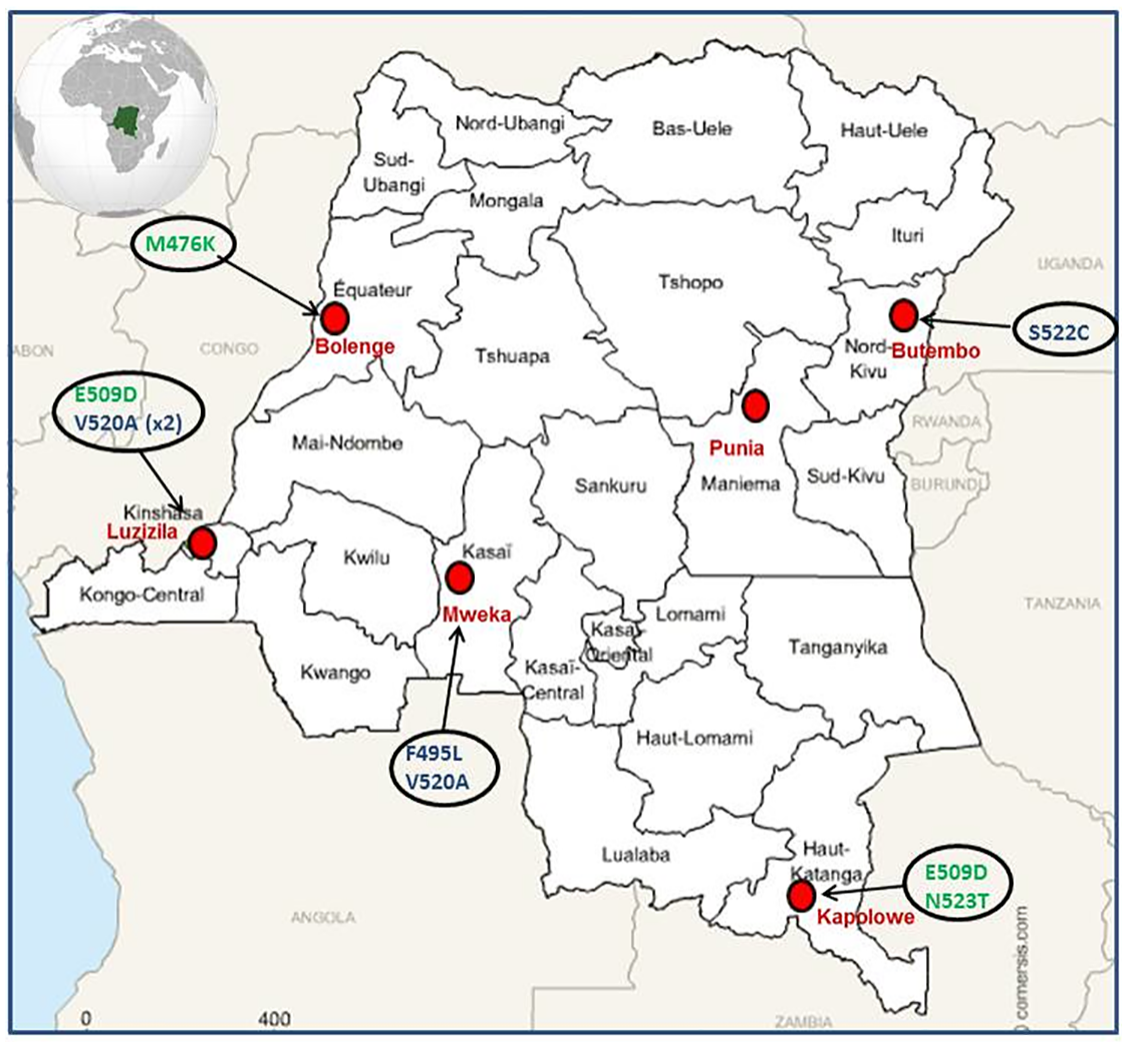
Distribution of mutations found in the k13 gene across DRC. New mutations are in green and already described mutations are presented in blue.

On the 280 samples analyzed, sequencing was correctly done for 261 samples. We identified 9 samples (3.4%) with mutations in the propeller domain of the K13 among which 3 mutations previously described (F495L, S522C and V520A) and 3 new mutations (M476K, N523T and E509D)(Fig 1).

## Discussion

Since the Congolese NMCP introduced ACT as first-line treatment in 2005, whatever some local studies have assessed ACT efficacy, only one national survey, conducted by the NMCP, has been performed to assess malaria first-line treatment efficacy. But results of this study have not yet been published.

In this study, we reported only 1% (3/280) of the samples that have increased *pfmdr1* copy number and all of these samples are from Butembo, in the east of the country. This is the first time that amplification of *pfmdr1* gene copy number is assessed in DRC. Our results are similar to those reported from African samples by Uhlemann et al. in Gabon [28], Ngalah et al. in Kenya [22] or Gadalla et al. [29] in Sudan where low prevalence (< 10%) of *pfmdr1 gene* copy number was detected. That seems to be different than in South-east Asia where amplification of *Pfmdr1* copy number is frequently high [21, 26, 30]. Duah et al. have incriminated the use of ACT in the occurrence of *P*. *falciparum* strains with *pfmdr1* amplification in African samples [31]. One other reason to explain this difference could be that mefloquine, a drug that selects lines with increased *pfmdr1* copy number [9], was widely used in Asia both in monotherapy and as partner drug in ACT, comparatively than in Africa.

Increased *pfmdr1* copy number has been linked with treatment failure (or reduced sensitivity) to some ACT [18, 21, 22, 29, 32]. The evidence for the first time of this increased *pfmdr1* copy number in the East of the DRC emphasizes the importance of maintaining regular monitoring.

As previously observed for Sulfadoxine-Pyrimethamine or chloroquine, *pfmdr1* resistance-based is maybe emerging in the East of the country before to expand to the rest of the country. Before the present work, only one study assessed *pfmdr1* polymorphism in DRC but by exploring the N86Y mutation [33].

Despite the fact that prevalence of the K76T mutation (related to chloroquine-resistance) is still in a high level in our results (65.7%) and that a cross resistance has been described between chloroquine and amodiaquine [34], the SVMNT haplotype linked to *in vivo* and *in vitro* amodiaquine resistance [35], has not been found in our samples. Currently, this haplotype has been reported to be present in only five African countries, including two bordering the DRC (Angola and Tanzania) [36–40]. It seems that this haplotype is quite rare in Africa compared to Asia. But continuous use of the artesunate-amodiaquine combination in DRC as first-line treatment could make selection for this resistant strain in the future.

After analysis of the *pfk13* gene, six different mutations in the propeller domain were detected into nine samples (3.2%). Two of these mutations have been previously described in other African countries. The S522C mutation was isolated in the present study in a sample from Butembo and has been previously reported in Uganda [41], which is relatively close to Butembo (~80 Km). Due to the important trade and the frequent movement of population between both countries around this geographic area, we can hypothesize that this mutant has disseminated, but it’s not excluded that this mutant has independently emerged in DRC.

On the other hand, we found the V520A mutation in two sites relatively distant (Luzizila and Mweka). Our results are similar to those reported by Taylor et al. who found this mutation in DRC too, in the same geographic area than us. In the results provided by Taylor et al. in 2015, this mutation appears to be the most common in African parasites as it has been reported in several African countries (Gambia, Mali, Ghana, Burkina-Faso, Kenya, Tanzania, Malawi and DRC) [41]. One must ask if this mutation has spread out from one point or if it has appeared spontaneously in many areas. Further phylogenetic analysis could put light on that. However, a recent published multi-countries study has not found this mutation but has reported the A578S mutation as the most prevalent in Africa [42]. We have not either found this mutation in our samples.

Surprisingly, the F495L mutation that we found in Mweka was described for the first time in samples from the China Myanmar border [43] then in Mayotte [44].

We also found three undescribed mutations on the *pfk13* gene (M476K, E509D and N523T). The M476K mutation could be of a particular interest because one mutation on this position, the M476I, that appeared *in vitro* after artemisinin pressure in an African line from Tanzania (F32-ART) [16, 45]. This M476I mutation was also largely found in isolates from Myanmar [46]. Unfortunately, we cannot presently define what are the clinical implications of this new M476K mutation.

The newly reported E509D and N523T mutations are not related or close to resistant polymorphisms described in Asia.

None of the mutations clearly correlated to increased parasite clearance time in Asian samples have been found in Africa yet.

## Conclusion

The data reported in this study reports the occurrence of some polymorphisms onto *P*. *falciparum* genes related to drug resistance. None of the mutations clearly associated to ACT resistance have been found in this study.

As in the rest of Africa, resistance to artemisinin seems not to be yet present there. DRC is one of the rare countries that have officially adopted multiple first-line treatment in its policy. This could provide a protective effect to the emergence of resistant strains but discovery of new mutations on the *pfk13* gene highlights the importance of a continuous monitoring. We unfortunately have not assessed in vitro susceptibility of the isolates with these new mutations.

## Supporting information

S1 Table Basic data related to individuals with *P*. *falciparum* positive samples. (DOCX)

## References

[1] World Health Organization. World Malaria Report. Geneva: WHO Press; 2015.

[2] WHO. Global technical strategy for malaria 2016–2030. Geneva, World Health Organization; 2015

[3] Bruce-Chwatt LJ, Black RH, Canfield CJ, Clyde DF, Peters W and Wernsdorfer WH.Chemotherapy of malaria. Geneva, World Health Organization, 1986.

[4] Pradines B, Dormoire J, Briolant S, Bogreau H, Rogier C.La résistance aux antipaludiques. Rev Fr Lab. 2010, 422: 51–62.

[5] WHO. Guidelines for the treatment of malaria. Third Edition Geneva, World Health Organization; 2015

[6] Murray CJ, Rosenfeld LC, Lim SS, Andrews KG, Foreman KJ, Haring D, et al Global malaria mortality between 1980 and 2010: a systematic analysis. Lancet 2012, 379:413–431. 22305225 10.1016/S0140-6736(12)60034-8

[7] Phillips M, Phillips-Howard PA. Economic implications of resistance to antimalarial drugs. Pharmacoeconomics 1996, 10:225–238. 10163571 10.2165/00019053-199610030-00004

[8] Talisuna AO, Bloland P, D’Alessandro U. History, dynamics, and public health importance of malaria parasite resistance. Clinical and Microbiology Reviews 2004, 17:235–254. 10.1128/CMR.17.1.235-254.2004 PMC321461 14726463

[9] Price RN, Nosten F. Drug resistant falciparum malaria: clinical consequences and strategies for prevention. Drug Resistance Updates 2001, 4:187–196. 10.1054/drup.2001.0195 11768332

[10] White NJ, Nosten F, Looareesuwan S, Watkins WM, Marsh K, Snow RW, et al Averting a malaria disaster. Lancet 1999, 353:1965–1967. 10371589 10.1016/s0140-6736(98)07367-x

[11] WHO. *Update on artemisinin and ACT resistance*, *April 2016*. Geneva, World Health Organization; 2016

[12] Plowe CV, Roper C, Barnwell JW, Happi CT, Joshi HH, Mbacham W, et al World Antimalarial Resistance Network (WARN) III: molecular markers for drug resistant malaria. Malar J. 2007;6:121 10.1186/1475-2875-6-121 17822535 PMC2008207

[13] Mvumbi DM, Kayembe N, Situakibanza NT, Bobanga L, Mvumbi L, De Mol P, et al Falciparum malaria molecular drug resistance in the Democratic Republic of Congo: a systematic review. Malar J 2015,14:354 10.1186/s12936-015-0892-z 26376639 PMC4574228

[14] Djimde A, Doumbo OK, Cortese JF, Kayentao K, Doumbo S, Diourte Y, et al A molecular marker for chloroquine-resistant falciparum malaria. N Engl J Med 2001;344:257–63 10.1056/NEJM200101253440403 11172152

[15] Sa JM, Twu O.Protecting the malaria drug arsenal: halting the rise and spread of amodiaquine resistance by monitoring the PfCRT SVMNT type. Malar J 2010, 9:374 10.1186/1475-2875-9-374 21182787 PMC3020158

[16] Ariey F, Witkowski B, Amaratunga C, Beghain J, Langlois AC, Khim N, et al A molecular marker of artemisinin-resistant Plasmodium falciparum malaria. Nature. 2014; 13:50–55. 10.1038/nature12876 PMC5007947 24352242

[17] Chavchich M, Gerena L, Peters J, Chen N, Cheng Q, Kyle DE. Role of pfmdr1 amplification and expression in induction of resistance to artemisinin derivatives in Plasmodium falciparum. Antimicrob Agents Chemother. 2010;54:2455–64. 10.1128/AAC.00947-09 20350946 PMC2876417

[18] Lobo E, de Sousa B, Rosa S, Figueiredo P, Lobo L, Pateira S, et al Prevalence of pfmdr1 alleles associated with artemether-lumefantrine tolerance/resistance in Maputo before and after the implementation of artemisinin-based combination therapy. Malar J. 2014;13:300 10.1186/1475-2875-13-300 25098280 PMC4248432

[19] Preechapornkul P, Imwong M, Chotivanich K, Pongtavornpinyo W, Dondorp AM, et al Plasmodium falciparum pfmdr1 *amplification*, *mefloquine resistance*, *and parasite fitness*. Antimicrob Agents Chemother. 2009;53:1509–15. 10.1128/AAC.00241-08 19164150 PMC2663078

[20] Sidhu AB, Uhlemann AC, Valderramos SG, Valderramos JC, Krishna S, Fidock DA. Decreasing pfmdr1 copy number in Plasmodium falciparum malaria heightens susceptibility to mefloquine, lumefantrine, halofantrine, quinine, and artemisinin. J Infect Dis. 2006;194:528–35. 10.1086/507115 16845638 PMC2978021

[21] Lim P, Alker AP, Khim N, Shah NK, Incardona S, Doung S, et al pfmdr1 copy number and arteminisin derivatives combination therapy failure in falciparum malaria in Cambodia. Malar J. 2009;8:11 10.1186/1475-2875-8-11 19138391 PMC2627910

[22] Ngalah BS, Ingasia LA, Cheruiyot AC, Chebon LJ, Juma DW, Muiruri P, et al Analysis of major genome loci underlying artemisinin resistance and pfmdr1 copy number in pre- and post-ACTs in western Kenya. Sci Rep 2015;5:8308 10.1038/srep08308 25655315 PMC4319159

[23] Mvumbi DM, Boreux R, Sacheli R, Lelo M, Lengu B, Nani-Tuma S, et al Assessment of pfcrt 72–76 haplotypes eight years after chloroquine withdrawal in Kinshasa. Democratic Republic of Congo. Malar J.2013;12:459 10.1186/1475-2875-12-459 24359280 PMC3878180

[24] Cnops L, Jacobs J and Van Esbroeck M. Validation of a four-primer real-time PCR as a diagnostic tool for single and mixed Plasmodium infections. ClinMicrobiol Infect 2011; 17: 1101–1107 10.1111/j.1469-0691.2010.03344.x 20718798

[25] WWARN. Copy number estimation of P. falciparum pfmdr1. Molecular Module 2011.

[26] Price RN, Uhlemann AC, Brockman A, McGready R, Ashley E, Phaipun L, et al Mefloquine resistance in Plasmodium falciparum and increased pfmdr1 gene copy number. Lancet. 2004; 364: 438–47. 10.1016/S0140-6736(04)16767-6 15288742 PMC4337987

[27] Mvumbi DM, Bobanga L, Melin P, De Mol P, Kayembe N, Situakibanza NT, et al High prevalence of Plasmodium falciparum infection in asymptomatic individuals from the Democratic Republic of Congo. Malar Res Treat 2016, Article ID 5405802. 10.1155/2016/5405802 26942036 PMC4749826

[28] Uhlemann AC, Ramharter M, Lell B, Kremsner PG, Krishna S. Amplification of Plasmodium falciparum multidrug resistance gene 1 in isolates from Gabon. J Infect Dis. 2005, 192: 1830–1835. 10.1086/497337 16235185

[29] Gadalla NB, Adam I, Elzaki SE, Bashir S, Mukhtar I, Oguike M, et al *Increased* pfmdr*1 copy number and sequence polymorphisms in* Plasmodium falciparum *isolates from Sudanese malaria patients treated with artemether-lumefantrine*. Antimicrob Agents Chemother. 2011;55:5408–5411. 10.1128/AAC.05102-11 21896916 PMC3195064

[30] Mungthin M, Khositnithikul R, Sitthichot N, Suwandittakul N, Wattanaveeradej V, Ward SA, et al Association between the pfmdr1 gene and in vitro artemether and lumefantrine sensitivity in Thai isolates of Plasmodium falciparum. Am J Trop Med Hyg. 2010;83:1005–1009. 10.4269/ajtmh.2010.10-0339 21036827 PMC2963959

[31] Duah NO, Matrevi SA, de Souza DK, Binnah DD, Tamakloe MM, Opoku VS, et al Increased pfmdr1 gene copy number and the decline in pfcrt and pfmdr1 resistance alleles in Ghanaian Plasmodium falciparumisolates after the change of anti-malarial drug treatment policy. Malar J. 2013;12:377 10.1186/1475-2875-12-377 24172030 PMC3819684

[32] Dokomajilar C, Nsobya SL, Greenhouse B, Rosenthal PJ, Dorsey G. Selection of Plasmodium falciparum pfmdr1 alleles following therapy with artemether-lumefantrine in an area of Uganda where malaria is highly endemic. Antimicrob Agents Chemother 2006; 50:1893–1895. 10.1128/AAC.50.5.1893-1895.2006 16641472 PMC1472234

[33] Mobula L, Lilley B, Tshefu AK, Rosenthal PJ.Resistance-mediating Polymorphisms in Plasmodium falciparum infections in Kinshasa, Democratic Republic of the Congo. Am J Trop Med Hyg 2009, 80:555–558. 19346374

[34] Ochong EO, van den Broek IV, Keus K, Nzila A. Short report: association between chloroquine and amodiaquine resistance and allelic variation in the Plasmodium falciparum multiple drug resistance 1 gene and the chloroquine resistance transporter gene in isolates from the upper Nile in southern Sudan. Am J Trop Med Hyg 2003, 69:184–187. 13677373

[35] Beshir K, Sutherland CJ, Merinopoulos I, Durrani N, Leslie T, Rowland M, et al Amodiaquine resistance in Plasmodium falciparum malaria in Afghanistan is associated with the pfcrt SVMNT allele at codons 72 to 76. Antimicrob Agents Hemother 2010, 54:3714–3716. 10.1128/AAC.00358-10 PMC2934991 20547800

[36] Alifrangis M, Dalgaard MB, Lusingu JP, Vestergaard LS, Staalsoe T, Jensen AT, et al Occurrence of the Southeast Asian/South American SVMNT haplotype of the chloroquine-resistance transporter gene in Plasmodium falciparum in Tanzania. J Infect Dis 2006, 193:1738–1741. 10.1086/504269 16703518

[37] Mehlotra RK, Mattera G, Bockarie MJ, Maguire JD, Baird JK, Sharma YD, et al Discordant patterns of genetic variation at two chloroquine resistance loci in worldwide populations of the malaria parasite Plasmodium falciparum. Antimicrob Agents Chemother 2008, 52:2212–2222. 10.1128/AAC.00089-08 18411325 PMC2415780

[38] Gama BE, de Carvalho GA P, LutucutaKosi FJ, de Oliveira NK A, Fortes F, et al Plasmodium falciparum isolates from Angola show the SVMNT haplotype in the pfcrt gene. Malar J 2010, 9:174 10.1186/1475-2875-9-174 20565881 PMC2898790

[39] Mekonnen SK, Aseffa A, Berhe N, Teklehaymanot T, Clouse RM, Gebru T, et al Return of chloroquine-sensitive Plasmodium falciparum parasites and emergence of chloroquine-resistant Plasmodium vivax in Ethiopia. Malar J. 2014;13:244 10.1186/1475-2875-13-244 24964730 PMC4230645

[40] Ngassa Mbenda HG, Das A. Occurrence of multiple chloroquine-resistant Pfcrt haplotypes and emergence of the S(agt) VMNT type in Cameroonian Plasmodium falciparum. J Antimicrob Chemother. 2014;69:400–3. 10.1093/jac/dkt388 24092656

[41] Taylor SM, Parobek CM, DeConti DK, Kayentao K, Coulibaly SO, Greenwood BM, et al Absence of putative artemisinin resistance mutations among Plasmodium falciparum in Sub-Saharan Africa: a molecular epidemiologic study. J Infect Dis 2015; 211: 680–8. 10.1093/infdis/jiu467 25180240 PMC4402372

[42] Ménard D, Khim N, Beghain J, Adegnika AA, Shafiul-Alam M, Amodu O, et al A Worldwide Map of Plasmodium falciparum K13-Propeller Polymorphisms. N Engl J Med 2016;374:2453–64 10.1056/NEJMoa1513137 27332904 PMC4955562

[43] Wang Z, Shrestha S, Li X, Miao J, Yuan L, Cabrera M, et al Prevalence of K13-propeller polymorphisms in Plasmodium falciparum from China-Myanmar border in 2007–2012. Malar J 2015; 14:168 10.1186/s12936-015-0672-9 PMC4404080 25927592

[44] Torrentino-Madamet M, Collet L, Lepère J, Benoit N, Amalvict R, Ménard D, et al. K13-propeller polymorphisms in Plasmodium falciparum isolates from patients in Mayotte in 2013 and 2014. Antimicrob Agents Chemother. 409 2015;59:7878–81. 10.1128/AAC.01251-15 26416865 PMC4649217

[45] Witkowski B, Lelièvre J, Barragán MJL, Laurent V, Su X, Berry A, et al. Increased tolerance to artemisinin in Plasmodium falciparum is mediated by a quiescence mechanism. Antimicrob Agents Chemother. 2010;54:1872–1877. 10.1128/AAC.01636-09 20160056 PMC2863624

[46] Nyunt MH, Hlaing T, Oo HW, Tin-Oo L-LK, Phway HP, Wang B, et al Molecular assessment of artemisinin resistance markers, polymorphisms in the K13 propeller, and a multidrug-resistance gene in the eastern and western border areas of Myanmar. Clin Infect Dis. 2015;60:1208–1215. 10.1093/cid/ciu1160 25537878

