# Supplementary material for: Molecular surveillance of Plasmodium falciparum resistance to artemisinin-based combination therapies in the Democratic Republic of Congo
Source: PLoS One. 2017 Jun 8;12(6):e0179142. doi: 10.1371/journal.pone.0179142 (PMC5464640; doi:10.1371/journal.pone.0179142)
Supplement: S1 Table — (DOCX) [file pone.0179142.s001.docx]

S1 Table. Basic data related to individuals with *P. falciparum* positive samples

| **N°** | **Age (yrs)** | **Sex** | **Localisation** | **Pfmdr1** | **Pfcrt** | **K13** |
| --- | --- | --- | --- | --- | --- | --- |
| 1 | 5 | F | Bolenge | W | CVIET | M (M476K) |
| 5 | 3 | M | Bolenge | W | CVMNK | W |
| 12 | 3 | F | Bolenge | W | CVIET | W |
| 35 | 58 | F | Bolenge | W | CVIET | W |
| 46 | 9 | F | Bolenge | W | CVMNK | W |
| 17 | 12 | M | Bolenge | W | CVIET | W |
| 27 | 32 | M | Bolenge | W | CVIET | W |
| 45 | 12 | M | Bolenge | W | CVMNK | W |
| 2 | 14 | M | Bolenge | W | CVIET | W |
| 3 | 1 | F | Bolenge | W | CVIET | W |
| 4 | 53 | M | Bolenge | W | CVIET | W |
| 6 | 5 | F | Bolenge | W | CVIET | N/A |
| 7 | 2 | F | Bolenge | W | CVIET | W |
| 8 | 3 | F | Bolenge | W | CVIET | W |
| 9 | 2 | F | Bolenge | W | CVIET | W |
| 10 | 4 | F | Bolenge | W | CVIET | W |
| 11 | 5 | F | Bolenge | W | CVIET | N/A |
| 13 | 10 | F | Bolenge | W | CVIET | W |
| 14 | 12 | F | Bolenge | W | CVIET | W |
| 15 | 12 | M | Bolenge | W | CVIET | W |
| 16 | 8 | F | Bolenge | W | CVIET | W |
| 18 | 13 | M | Bolenge | W | CVIET | W |
| 19 | 13 | M | Bolenge | W | CVIET | N/A |
| 20 | 15 | F | Bolenge | W | CVIET | W |
| 21 | 8 | F | Bolenge | W | CVIET | W |
| 22 | 59 | M | Bolenge | W | CVIET | W |
| 23 | 55 | M | Bolenge | W | CVIET | W |
| 24 | 41 | M | Bolenge | W | CVIET | W |
| 25 | 42 | F | Bolenge | W | CVIET | W |
| 26 | 19 | M | Bolenge | W | CVIET | W |
| 28 | 47 | F | Bolenge | W | CVIET | W |
| 29 | 29 | F | Bolenge | W | CVIET | W |
| 30 | 30 | F | Bolenge | W | CVIET | W |
| 31 | 29 | M | Bolenge | W | CVIET | W |
| 32 | 33 | F | Bolenge | W | CVIET | W |
| 33 | 41 | F | Bolenge | W | CVIET | W |
| 34 | 53 | F | Bolenge | W | CVIET | W |
| 36 | 20 | F | Bolenge | W | CVIET | W |
| 37 | 1 | F | Bolenge | W | CVMNK | W |
| 38 | 2 | M | Bolenge | W | CVMNK | W |
| 39 | 3 | F | Bolenge | W | CVMNK | W |
| 40 | 5 | F | Bolenge | W | CVMNK | W |
| 41 | 4 | F | Bolenge | W | CVMNK | W |
| 42 | 12 | F | Bolenge | W | CVMNK | W |
| 43 | 13 | F | Bolenge | W | CVMNK | W |
| 44 | 11 | F | Bolenge | W | CVMNK | W |
| 47 | 19 | M | Bolenge | W | CVMNK | W |
| 48 | 57 | F | Bolenge | W | CVMNK | W |
| 49 | 58 | F | Bolenge | W | CVMNK | W |
| 50 | 29 | F | Bolenge | W | CVMNK | W |
| 51 | 18 | M | Bolenge | W | CVMNK | W |
| 52 | 21 | F | Butembo | W | CVMNK | M (S522C) |
| 53 | 49 | F | Butembo | W | CVIET | N/A |
| 54 | 34 | F | Butembo | W | CVIET | W |
| 55 | 35 | F | Butembo | W | CVIET | W |
| 56 | 48 | F | Butembo | W | CVIET | W |
| 57 | 52 | F | Butembo | W | CVIET | W |
| 58 | 20 | F | Butembo | W | CVIET | W |
| 59 | 6 | F | Butembo | W | CVIET | W |
| 60 | 13 | F | Butembo | W | CVIET | W |
| 61 | 7 | F | Butembo | W | CVIET | W |
| 62 | 9 | F | Butembo | W | CVIET | W |
| 63 | 1 | M | Butembo | W | CVIET | W |
| 64 | 48 | F | Butembo | M | CVIET | W |
| 65 | 19 | M | Butembo | M | CVIET | W |
| 66 | 39 | F | Butembo | W | CVMNK | W |
| 67 | 24 | M | Butembo | W | CVMNK | W |
| 68 | 36 | F | Butembo | W | CVMNK | W |
| 69 | 33 | F | Butembo | W | CVMNK | W |
| 70 | 48 | F | Butembo | W | CVMNK | W |
| 71 | 7 | F | Butembo | W | CVMNK | W |
| 72 | 3 | M | Butembo | W | CVMNK | W |
| 73 | 58 | F | Butembo | M | CVMNK | W |
| 74 | 27 | F | Kapolowe | W | CVIET | M (E509D) |
| 75 | 52 | M | Kapolowe | W | CVIET | M (N523T) |
| 107 | 36 | M | Kapolowe | W | CVIET | N/A |
| 108 | 24 | F | Kapolowe | W | CVIET | N/A |
| 117 | 2 | M | Kapolowe | W | CVMNK | N/A |
| 130 | 49 | F | Kapolowe | W | CVMNK | N/A |
| 76 | 11 | F | Kapolowe | W | CVIET | W |
| 77 | 58 | M | Kapolowe | W | CVIET | W |
| 78 | 50 | F | Kapolowe | W | CVMNK | W |
| 79 | 3 | M | Kapolowe | W | CVIET | W |
| 80 | 1 | F | Kapolowe | W | CVIET | W |
| 81 | 5 | M | Kapolowe | W | CVIET | W |
| 82 | 2 | M | Kapolowe | W | CVIET | W |
| 83 | 1 | M | Kapolowe | W | CVIET | W |
| 84 | 3 | F | Kapolowe | W | CVIET | W |
| 85 | 3 | F | Kapolowe | W | CVIET | W |
| 86 | 3 | F | Kapolowe | W | CVIET | W |
| 87 | 1 | F | Kapolowe | W | CVIET | W |
| 88 | 10 | M | Kapolowe | W | CVIET | W |
| 89 | 15 | F | Kapolowe | W | CVIET | W |
| 90 | 8 | F | Kapolowe | W | CVIET | W |
| 91 | 6 | F | Kapolowe | W | CVIET | W |
| 92 | 14 | M | Kapolowe | W | CVIET | W |
| 93 | 15 | M | Kapolowe | W | CVIET | W |
| 94 | 11 | F | Kapolowe | W | CVIET | W |
| 95 | 13 | F | Kapolowe | W | CVIET | W |
| 96 | 10 | M | Kapolowe | W | CVIET | W |
| 97 | 8 | F | Kapolowe | W | CVIET | W |
| 98 | 9 | M | Kapolowe | W | CVIET | W |
| 99 | 14 | F | Kapolowe | W | CVIET | W |
| 100 | 11 | F | Kapolowe | W | CVIET | W |
| 101 | 7 | F | Kapolowe | W | CVIET | W |
| 102 | 13 | F | Kapolowe | W | CVIET | W |
| 103 | 46 | M | Kapolowe | W | CVIET | W |
| 104 | 9 | M | Kapolowe | W | CVIET | W |
| 105 | 34 | M | Kapolowe | W | CVIET | W |
| 106 | 16 | F | Kapolowe | W | CVIET | W |
| 109 | 16 | F | Kapolowe | W | CVIET | W |
| 110 | 56 | M | Kapolowe | W | CVIET | W |
| 111 | 49 | F | Kapolowe | W | CVIET | W |
| 112 | 36 | F | Kapolowe | W | CVIET | W |
| 113 | 30 | M | Kapolowe | W | CVIET | W |
| 114 | 64 | F | Kapolowe | W | CVIET | W |
| 115 | 35 | M | Kapolowe | W | CVIET | W |
| 116 | 9 | M | Kapolowe | W | CVIET | W |
| 118 | 1 | F | Kapolowe | W | CVMNK | W |
| 119 | 1 | F | Kapolowe | W | CVMNK | W |
| 120 | 5 | M | Kapolowe | W | CVMNK | W |
| 121 | 6 | F | Kapolowe | W | CVMNK | W |
| 122 | 10 | M | Kapolowe | W | CVMNK | W |
| 123 | 9 | M | Kapolowe | W | CVMNK | W |
| 124 | 11 | F | Kapolowe | W | CVMNK | W |
| 125 | 12 | M | Kapolowe | W | CVMNK | W |
| 126 | 12 | F | Kapolowe | W | CVMNK | W |
| 127 | 11 | F | Kapolowe | W | CVMNK | W |
| 128 | 15 | F | Kapolowe | W | CVMNK | W |
| 129 | 19 | M | Kapolowe | W | CVMNK | W |
| 131 | 59 | M | Kapolowe | W | CVMNK | W |
| 132 | 56 | F | Kapolowe | W | CVMNK | W |
| 133 | 34 | F | Kapolowe | W | CVMNK | W |
| 134 | 48 | F | Kapolowe | W | CVMNK | W |
| 135 | 72 | M | Kapolowe | W | CVMNK | W |
| 136 | 60 | F | Kapolowe | W | CVMNT | W |
| 137 | 39 | M | Luzizila | W | CVIET | M (E509D) |
| 138 | 46 | F | Luzizila | W | CVIET | M (V520A) |
| 139 | 24 | F | Luzizila | W | CVIET | M (V520A) |
| 142 | 12 | F | Luzizila | W | CVIET | N/A |
| 144 | 56 | F | Luzizila | W | CVMNK | N/A |
| 146 | 11 | F | Luzizila | W | CVMNK | N/A |
| 159 | 19 | F | Luzizila | W | CVIET | N/A |
| 175 | 53 | F | Luzizila | W | CVMNK | N/A |
| 185 | 23 | F | Luzizila | W | CVMNK | N/A |
| 188 | 44 | F | Luzizila | W | CVMNK | N/A |
| 140 | 27 | F | Luzizila | W | CVIET | W |
| 141 | 59 | F | Luzizila | W | CVIET | W |
| 143 | 41 | F | Luzizila | W | CVMNK | W |
| 145 | 40 | M | Luzizila | W | CVMNK | W |
| 147 | 21 | M | Luzizila | W | CVIET | W |
| 148 | 43 | F | Luzizila | W | CVIET | W |
| 149 | 23 | F | Luzizila | W | CVIET | W |
| 150 | 39 | F | Luzizila | W | CVIET | W |
| 151 | 44 | F | Luzizila | W | CVIET | W |
| 152 | 30 | F | Luzizila | W | CVIET | W |
| 153 | 18 | F | Luzizila | W | CVIET | W |
| 154 | 58 | F | Luzizila | W | CVIET | W |
| 155 | 35 | F | Luzizila | W | CVIET | W |
| 156 | 16 | F | Luzizila | W | CVIET | W |
| 157 | 16 | F | Luzizila | W | CVIET | W |
| 158 | 37 | F | Luzizila | W | CVIET | W |
| 160 | 33 | M | Luzizila | W | CVIET | W |
| 161 | 22 | F | Luzizila | W | CVIET | W |
| 162 | 56 | M | Luzizila | W | CVIET | W |
| 163 | 21 | F | Luzizila | W | CVIET | W |
| 164 | 31 | F | Luzizila | W | CVIET | W |
| 165 | 38 | F | Luzizila | W | CVIET | W |
| 166 | 40 | F | Luzizila | W | CVIET | W |
| 167 | 53 | F | Luzizila | W | CVIET | W |
| 168 | 49 | F | Luzizila | W | CVIET | W |
| 169 | 9 | F | Luzizila | W | CVIET | W |
| 170 | 13 | M | Luzizila | W | CVIET | W |
| 171 | 15 | F | Luzizila | W | CVIET | W |
| 172 | 3 | F | Luzizila | W | CVIET | W |
| 173 | 3 | F | Luzizila | W | CVIET | W |
| 174 | 1 | F | Luzizila | W | CVIET | W |
| 176 | 32 | M | Luzizila | W | CVMNK | W |
| 177 | 44 | F | Luzizila | W | CVMNK | W |
| 178 | 39 | F | Luzizila | W | CVMNK | W |
| 179 | 29 | F | Luzizila | W | CVMNK | W |
| 180 | 45 | M | Luzizila | W | CVMNK | W |
| 181 | 19 | F | Luzizila | W | CVMNK | W |
| 182 | 56 | F | Luzizila | W | CVMNK | W |
| 183 | 59 | M | Luzizila | W | CVMNK | W |
| 184 | 24 | M | Luzizila | W | CVMNK | W |
| 186 | 16 | F | Luzizila | W | CVMNK | W |
| 187 | 33 | M | Luzizila | W | CVMNK | W |
| 189 | 48 | F | Luzizila | W | CVMNK | W |
| 190 | 6 | F | Luzizila | W | CVMNK | W |
| 191 | 12 | M | Luzizila | W | CVMNK | W |
| 192 | 11 | M | Luzizila | W | CVMNK | W |
| 193 | 13 | F | Luzizila | W | CVMNK | W |
| 194 | 13 | F | Luzizila | W | CVMNK | W |
| 195 | 2 | M | Luzizila | W | CVMNK | W |
| 196 | 5 | F | Luzizila | W | CVMNK | W |
| 197 | 4 | M | Luzizila | W | CVMNK | W |
| 198 | 3 | F | Luzizila | W | CVMNK | W |
| 199 | 19 | F | Mweka | W | CVIET | M (F495L) |
| 200 | 42 | F | Mweka | W | CVIET | M (V520A) |
| 201 | 33 | M | Mweka | W | CVMNK | N/A |
| 202 | 22 | M | Mweka | W | CVIET | W |
| 203 | 40 | F | Mweka | W | CVIET | W |
| 204 | 23 | F | Mweka | W | CVIET | W |
| 205 | 32 | F | Mweka | W | CVIET | W |
| 206 | 42 | M | Mweka | W | CVIET | W |
| 207 | 55 | F | Mweka | W | CVIET | W |
| 208 | 44 | F | Mweka | W | CVIET | W |
| 209 | 58 | F | Mweka | W | CVIET | W |
| 210 | 19 | M | Mweka | W | CVIET | W |
| 211 | 42 | F | Mweka | W | CVIET | W |
| 212 | 14 | F | Mweka | W | CVIET | W |
| 213 | 10 | F | Mweka | W | CVIET | W |
| 214 | 14 | F | Mweka | W | CVIET | W |
| 215 | 8 | F | Mweka | W | CVIET | W |
| 216 | 4 | F | Mweka | W | CVIET | W |
| 217 | 30 | M | Mweka | W | CVMNK | W |
| 218 | 43 | F | Mweka | W | CVMNK | W |
| 219 | 55 | M | Mweka | W | CVMNK | W |
| 220 | 26 | F | Mweka | W | CVMNK | W |
| 221 | 24 | M | Mweka | W | CVMNK | W |
| 222 | 51 | F | Mweka | W | CVMNK | W |
| 223 | 27 | F | Mweka | W | CVMNK | W |
| 224 | 2 | F | Mweka | W | CVMNK | W |
| 225 | 2 | F | Mweka | W | CVMNK | W |
| 226 | 14 | F | Mweka | W | CVMNK | W |
| 227 | 73 | F | Mweka | W | CVMNK | W |
| 228 | 66 | F | Mweka | W | CVMNK | W |
| 229 | 15 | F | Mweka | W | CVMNT | W |
| 230 | 40 | F | Punia | W | CVIET | N/A |
| 232 | 34 | F | Punia | W | CVMNK | N/A |
| 241 | 40 | F | Punia | W | CVIET | W |
| 231 | 40 | F | Punia | W | CVIET | W |
| 233 | 29 | F | Punia | W | CVIET | W |
| 234 | 54 | M | Punia | W | CVIET | W |
| 235 | 25 | M | Punia | W | CVIET | W |
| 236 | 58 | F | Punia | W | CVIET | W |
| 237 | 28 | M | Punia | W | CVIET | W |
| 238 | 21 | M | Punia | W | CVIET | W |
| 239 | 31 | F | Punia | W | CVIET | W |
| 240 | 33 | F | Punia | W | CVIET | W |
| 242 | 58 | F | Punia | W | CVIET | W |
| 243 | 37 | F | Punia | W | CVIET | W |
| 244 | 38 | M | Punia | W | CVIET | W |
| 245 | 55 | F | Punia | W | CVIET | W |
| 246 | 42 | F | Punia | W | CVIET | W |
| 247 | 24 | F | Punia | W | CVIET | W |
| 248 | 54 | F | Punia | W | CVIET | W |
| 249 | 29 | F | Punia | W | CVIET | W |
| 250 | 22 | F | Punia | W | CVIET | W |
| 251 | 17 | F | Punia | W | CVIET | W |
| 252 | 47 | F | Punia | W | CVIET | W |
| 253 | 22 | M | Punia | W | CVIET | W |
| 254 | 37 | F | Punia | W | CVIET | W |
| 255 | 33 | F | Punia | W | CVIET | W |
| 256 | 6 | F | Punia | W | CVIET | W |
| 257 | 10 | M | Punia | W | CVIET | W |
| 258 | 67 | F | Punia | W | CVIET | W |
| 259 | 72 | F | Punia | W | CVIET | W |
| 260 | 72 | F | Punia | W | CVIET | W |
| 261 | 60 | F | Punia | W | CVIET | W |
| 262 | 2 | F | Punia | W | CVIET | W |
| 263 | 1 | F | Punia | W | CVIET | W |
| 264 | 18 | M | Punia | W | CVMNK | W |
| 265 | 53 | F | Punia | W | CVMNK | W |
| 266 | 16 | M | Punia | W | CVMNK | W |
| 267 | 28 | F | Punia | W | CVMNK | W |
| 268 | 19 | F | Punia | W | CVMNK | W |
| 269 | 22 | M | Punia | W | CVMNK | W |
| 270 | 29 | M | Punia | W | CVMNK | W |
| 271 | 29 | F | Punia | W | CVMNK | W |
| 272 | 25 | M | Punia | W | CVMNK | W |
| 273 | 49 | F | Punia | W | CVMNK | W |
| 274 | 58 | M | Punia | W | CVMNK | W |
| 275 | 12 | M | Punia | W | CVMNK | W |
| 276 | 8 | F | Punia | W | CVMNK | W |
| 277 | 8 | F | Punia | W | CVMNK | W |
| 278 | 12 | M | Punia | W | CVMNK | W |
| 279 | 3 | M | Punia | W | CVMNK | W |
| 280 | 2 | F | Punia | W | CVMNK | W |
